# Access to Assistive Technology during the COVID-19 Global Pandemic: Voices of Users and Families

**DOI:** 10.3390/ijerph182111273

**Published:** 2021-10-27

**Authors:** Natasha Layton, Daniel Mont, Louise Puli, Irene Calvo, Kylie Shae, Emma Tebbutt, Keith D. Hill, Libby Callaway, Diana Hiscock, Abner Manlapaz, Inge Groenewegen, Mahpekai Sidiqi

**Affiliations:** 1 Rehabilitation Ageing and Independent Living (RAIL) Research Centre, Monash University, Melbourne 3800, Australia; keith.hill@monash.edu (K.D.H.); libby.callaway@monash.edu (L.C.); 2 Center for Inclusive Policy, Washington, DC 20005, USA; daniel.mont@inclusive-policy.org (D.M.); abner.manlapaz@inclusive-policy.org (A.M.); 3 Access to Assistive Technology Team, World Health Organization, 1211 Geneva, Switzerland; pulil@who.int (L.P.); calvoi@who.int (I.C.); shaek@who.int (K.S.); tebbute@who.int (E.T.); 4 Occupational Therapy Department, Monash University, Melbourne 3800, Australia; 5 HelpAge International, London SE1 7RL, UK; diana.hiscock@helpage.org; 6 Liliane Foundation, 5211 TX ‘s-Hertogenbosch, The Netherlands; IGroenewegen@lilianefonds.nl; 7 Kabul Orthopedic Organization, Kabul 1001, Afghanistan; mahpekay.sidiqy@yahoo.com

**Keywords:** assistive technology, policy, disability, ageing, assistive products, personnel, service provision, health systems, COVID-19, health workforce, universal health coverage

## Abstract

The SARS COVID-19 pandemic emerged in 2019 and has impacted people everywhere. Disparities in impact and outcomes are becoming apparent for individuals and communities which go beyond the trajectory of the disease itself, influenced by the strength and weaknesses of systems of universal health care, and the actions of civil society and government. This article is one of a series exploring COVID-19-related experiences of assistive technology (AT) users across the globe and implications for AT systems strengthening. AT such as mobility products, braille devices, and information communication technologies are key enablers of functioning, necessary to the achievement of the UN Sustainable Development Goals and enshrined in the Convention on the Rights of Persons with Disabilities. Reporting on a survey of 73 AT users across six global regions, we demonstrate that minority groups already living with health inequities are unduly impacted. An AT ecosystem analysis was conducted using the WHO GATE 5P framework, that is, people, products, personnel, provision and policy. AT users and families call for inclusive pandemic responses which encompass their needs across the lifespan, from very young to very old. We offer specific recommendations for future action to strengthen access to AT across public policy and civil society in pandemic preparedness and response.

## 1. Introduction

This research investigates the impact of a pandemic catastrophe upon users of assistive technology (AT). AT is an umbrella term which refers to assistive products and the related systems and services for their safe and effective use [1]. AT such as prosthetics, hearing aids, and communication devices enable independence and participation where functioning is affected by illness, disability or the impacts of ageing, and where environments present barriers [2,3].

It is estimated that less than one in ten people globally have access to the AT they need [4]. Multiple inequalities can compound this lack of access [5,6]. Factors including stigma, inaccessible environments and workplaces, poor access to education and healthcare can contribute to the experience of disadvantage [3,7,8,9]. AT users are likely to be specifically disadvantaged through the impacts of COVID-19 and require tailored policy responses [10,11].

An AT ecosystem must be present for the safe and effective provision and use of AT, to ensure that products are available, fit for purpose and the user’s environment, can be maintained, repaired and replaced as a person’s needs change. A process of selection, fitting, use and follow-up is needed to match assistive products to the person. A lack of these service steps leads to inefficient provision and poorer outcomes [12,13,14].

Responding to the mandate of the World Health Assembly Resolution on improving access to AT (WHA71.8) [15], the WHO is leading a range of AT system strengthening initiatives to support national and international policy responses. These responses are structured against a “5P Framework” representing five interlinked areas: people (AT users) [16], products (assistive products) [17], provision (service delivery) [1], personnel (workforce) [18] and policy [19]. Conceptualizing the AT system in this way provides a useful framework to map the landscape of AT access [7,20,21].

Then came COVID-19.

A novel coronavirus disease emerged in 2019 and a global pandemic was declared in early 2020 [22]. The repercussions of the pandemic are felt far beyond the primary impacts of the infection for those who contract it. Public health responses by communities, countries and regions to manage the pandemic have had multiple secondary consequences such as the disruption of global health, trade and travel, the derailment of the GDP of nation states and their capacity to pursue global health goals [23]. The emerging literature raises the alarm regarding accentuated health disparities and higher risks for specific populations across countries [24,25,26,27,28,29]; for specific age groups such as older people [29], children and adolescents [30]; people with specific conditions or impairments such as spinal cord injury [31], low vision and blindness [32]; and people pursuing specific activities such as students with disabilities [33,34].

This article is one in a series of three, exploring the effects of COVID-19 on AT users and their families [35] and on AT service providers [36]. Taking an AT ecosystem approach, this paper aims to investigate:

1. What are the experiences of AT users during the COVID-19 pandemic in relation to AT access and service delivery?
2. What are the implications of the COVID-19 pandemic for AT systems strengthening?

## 2. Methods

### 2.1. Research Design

**Figure 1:**
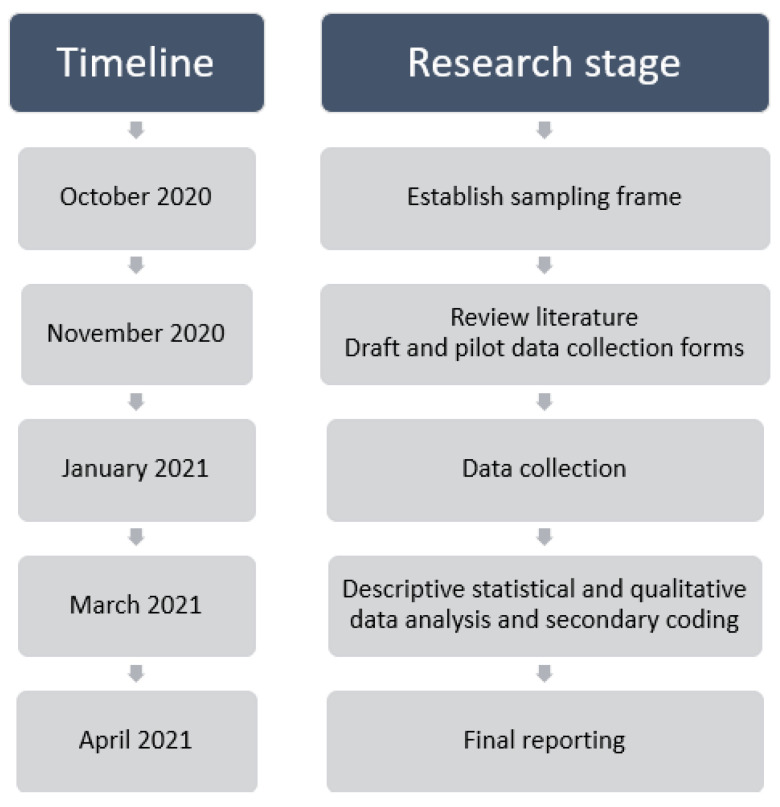
Summary research timeline and stages.

This qualitative study (see Figure 1) was designed according to COREQ qualitative guidelines [37].

A global research team was assembled including experts in providing or using assistive technology, disability and policy research. This team comprised researchers from two academic institutions, two international non-governmental organisations, and from WHO’s assistive technology team. The team utilised the literature (for a summary of the rapid evidence review, see Appendix A) and their experiences to develop a survey tool comprising closed and open questions (available in accessible electronic or paper formats) to explore the effects of COVID-19 on access to AT.

### 2.2. Sample

The research team used existing global, regional and national networks to share information about the research and calls for involvement with AT, health, disability and ageing entities across government and civil society. The survey invitation was widely distributed via email to 69 AT organisations in WHO’s register of global networks, and through the networks of each regional researcher. Organizations contacted were asked to share the survey invitations with their networks also in a snowball approach. The resulting participant sample of AT users and their families were mapped to each of the six WHO Regionsto recruit broad representation across contexts (urban/rural settings and World Bank income status), types of AT use, age and gender.

### 2.3. Data Collection

The survey tool was available in paper based and electronic formats, including an explanatory statement and consent section (survey questions reported in Appendix B). The paper-based survey could be completed using pen/pencil. The electronic survey complied with Web Content Accessibility Guidelines (www.w3.org/WAI/standards-guidelines/wcag/ (accessed on 22 August 2021)) and was able to be filled in by switch access users, or users of screen-reading software. All formats offered participants the option of identifiability or anonymity, with a default to anonymity if not explicit. A request to disseminate invitations to participate was sent by email. Recipients could consent to participate by returning the signed consent form or contact the principal investigator if they had questions. Surveys could be completed by respondents directly; civil society volunteers were available to translate or to scribe a person’s responses; or family members completed the survey on behalf of family members who used AT, including children. Completed surveys were emailed to the WHO team and uploaded to a secure password protected online folder on WHO’s server. Data were handled and retained in accordance with Monash University ethical requirements. All surveys collected between 1 December 2020 and 5 March 2021 were included.

### 2.4. Data Analysis

#### 2.4.1. Managing the Data

Qualitative and quantitative data were manually entered into Microsoft Excel Office 365 (Microsoft Corporation, North Sydney, Australia). Accuracy of data entry was confirmed through systematic line-by-line matching of paper and electronic versions. Respondent demographics were summarised using descriptive statistics appropriate to the data type. Qualitative data from the open-ended survey questions was uploaded into NVivo v11 (QRS International, Melbourne, Australia) for analysis.

#### 2.4.2. Approach to Analysis

Descriptive statistics were used to report upon the respondents. Thematic analysis of qualitative data provided a contextualised method to examine and understand participant experiences and perspectives within the context of environmental and societal impacts [38]. Codebook thematic analysis (analysing data using a set of themes derived from the literature), as well as reflexive coding to generate any additional themes were used [39]. Qualitative rigor was assured through dual coding by multiple researchers, bracketing through reflective fieldnotes, and consensus meetings to discuss emerging findings and reflections [40].

#### 2.4.3. Analysis Process

Two researchers undertook independent line-by-line coding to desegregate the text of each survey. Text describing similar phenomena were categorised into nodes from which themes and subthemes were developed. Two additional researchers each then independently coded subsets of data pertaining to older persons (15% or n-11), and data pertaining to significant disability (21% or n-16) and a consensus thematic meeting was held. The themes and subthemes were then presented to the primary research team once 50% of coding had taken place, proposing a coding tree along with illustrative examples of first-person quotes. Disagreements with the interpretation were resolved by discussion over a number of meetings until consensus was reached. The final theme and subthemes were presented in the results narrative with illustrative first-person quotes to support and evidence the interpretation. Article drafts were reviewed by all contributing authors.

## 3. Results

### 3.1. Descriptive Statistics

The sample (n-73) included 35 men and boys, and 38 women and girls, from infants to people over 90 years of age. Children (aged < 15) numbered n-28; adults of working age (15–64) numbered n-27; and older persons (60+) numbered n-18. In describing their lives and the reasons for AT use, many participants self-identified a range of body structure and function impairments including limb loss (n-13); polio (n-5), neurological impairments such as acquired brain injury, spinal cord injury and stroke (n-6); orthopedic issues such as club foot and osteoarthritis (n-5); cerebral palsy (n-9), developmental disability (n-6); hearing loss/deafness (n-11); vision loss/blindness (n-4); and multiple medical problems with associated functional limitations (n-10).

**Figure 2:**
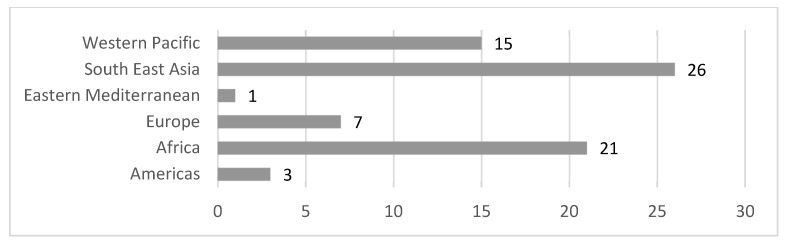
Number of AT user/family snapshots by WHO region.

Respondents were drawn from across world regions across 24 low-, middle- and high-income countries (see Figure 2).

**Figure 3:**
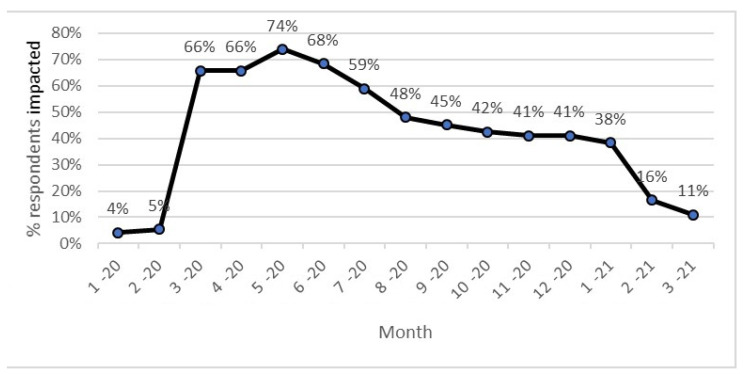
Months of COVID-19 impact.

The time horizon for this study were January 2020 through to March 2021. Impact was defined as the wider impact of the pandemic within the individual’s community on their AT use, not on whether or not the individual had contracted COVID-19. Figure 3 demonstrates the percentage of respondents reporting COVID-19-related impacts upon their communities monthly across this period. Factors reported as contributing to reduced impact over time, despite the ongoing presence of the COVID-19 pandemic are reported in the qualitative results.

**Figure 4:**
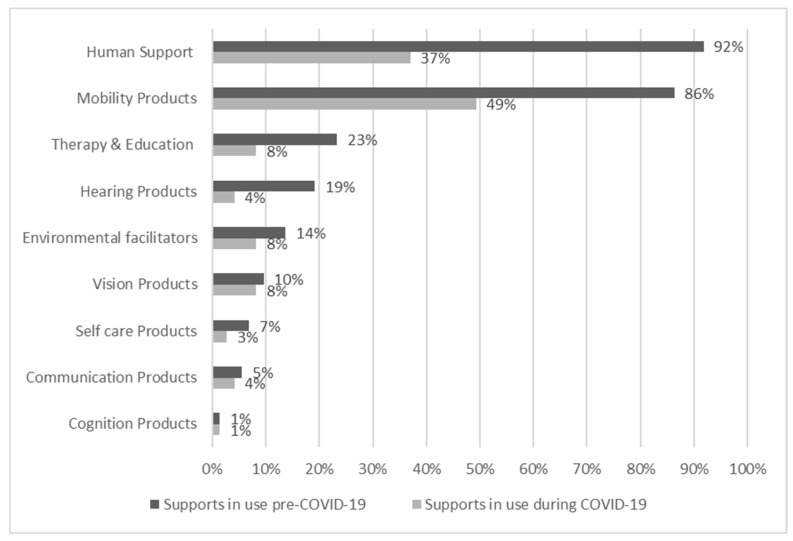
Percentage of people using various supports before and during COVID-19.

The survey asked participants what assistive products and services they used at two time points, before and then during COVID-19. Figure 4 displays this data organised according to the Washington Group on Disability Statistics (www.washingtongroup-disability.com (accessed on 22 August 2021)) six functioning domains (mobility, hearing, vision, self-care, communication, cognition) as well as additional areas of therapy and education, the environment, and human support.

Pre COVID-19, all respondents used more than one assistive product, with 50% of respondents identifying between one and three products (n-46) up to 10 + products (n-3). Human support, either paid, or unpaid such as from family, was the most common (67 instances), followed by assistive products for mobility such as prosthetics; walking aids or adapted bicycles (63 instances); therapy and educational supports (17 instances); products for hearing such as hearing aids and closed captioning (14); environmental facilitators including accessible public transport, accessible homes and workplaces (10); products for vision such as eye glasses, braille writing equipment, white cane, screen reading software and tactile markers (7); self-care products including incontinence and bathing supports (5); communication supports such as adapted computers, communication boards (4); and cognition support such as adapted abacus (1).

Participants described engaging in a broad range of activities as defined by WHO International Classification of Function (ICF), all enabled by combinations of AT, personal support, and environmental facilitators.

Before the pandemic, 47 people (nearly 64%) identified unmet need for AT and related support services, and this impacted both activities and participation. Participants described 188 individual supports in use prior to COVID-19, in contrast to 90 individual supports during COVID-19. Figure 4 depicts a significant overall decrease in the use of support during COVID-19.

Narratives within this section of the survey attributed decreased use to lack of products and constrained provision (e.g., a fall in availability of supports, particularly replacing old or outgrown products, and an inability to source parts); personnel (e.g., difficulty sourcing adequate human support for assessment, maintenance and troubleshooting), and policy challenges (e.g., closure or loss of related environmental and community supports.

These findings, taken together, indicate a decrease in availability of support as a result of COVID-19. The value of mixed methods research has been important here as the authors anticipate that, for some respondents, the survey design (where a person was invited to list their supports before and during COVID-19 in two separate questions) may have caused survey fatigue or burden.

### 3.2. Qualitative Results

**Figure 5:**
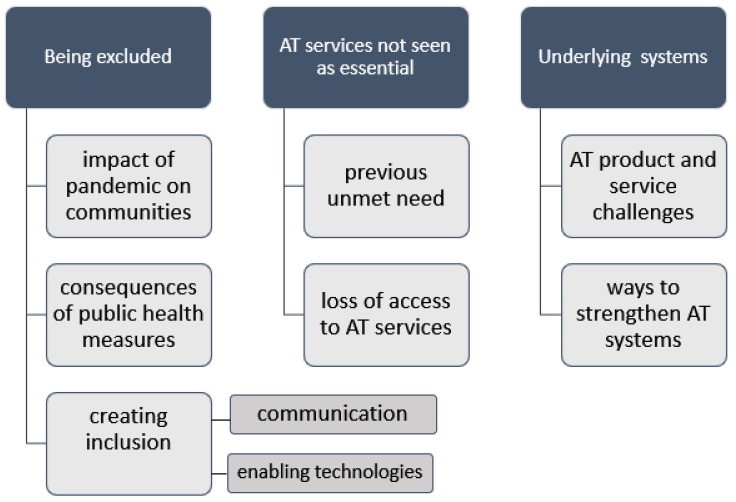
Thematic tree of experiences of AT users during the COVID-19 pandemic.

Before COVID-19, all respondents described lives enabled by AT and related supports. The experiences of AT users during the COVID-19 pandemic fell under three broad themes of being excluded from pandemic responses (the impact of pandemic on communities; the consequences of public health measures; and creating inclusion through communication and through enabling technologies); AT services not seen as essential by governments (previously unmet need; loss of access to AT services) and finally, underlying weakness of AT systems (AT product and service challenges, and ways to strengthen AT systems) (see Figure 5). Respondents identified a range of ideas and solutions, contributing to a set of recommendations for AT systems strengthening and these are explored below.

#### 3.2.1. Being Excluded

**Exclusion** can be defined as lack or denial of resources, rights, and the inability to participate alongside the majority of people in a community. Arguably, pandemic restrictions meant that all persons were equally excluded. However, while the experience of exclusion was perhaps equal, the ramifications of exclusion for AT users were amplified. Respondents described the impacts of exclusion from critical schooling and habilitation upon their mental and physical health,

*Due to COVID-19, the close down of therapy service physically, (his) condition deteriorated.* (SU51, Bangladesh)

*Field worker could no longer hold and play with me.* (SU40, Cameroon)

The overriding personal experience of COVID-19 consequences were expressed in terms of **fear**, specifically fear of infection for self or others, fear of repercussions when AT users found it untenable to adhere to restrictions, and community fear.

The real **risk of infection** with COVID-19 was, ‘*sad and traumatizing*’ (SU52, Malawi) and forty-two (57%) respondents felt at risk of getting COVID-19, ‘*It impacted in bad way. Neighbors died of COVID-19*’ (SU28, Ukraine). Thirty respondents (41%) stated important people around them were getting COVID-19 and feared going out of the home to support others or to attend school, church, mosque or hospital, and particularly when others ‘*didn’t maintain social distancing*’ (SU41, Bangladesh) or were ‘*not wearing masks*’ (SU13, UK). Unavoidable community contact was of concern for example to ‘*instruct the farm hired workers*’ (SU03, Kenya), or ‘*go get groceries or to work*’ (SU19, Kenya).

Respondents reported some occasions of community fear and mistrust, usually where having a disability appeared to be conflated with the risk of catching COVID-19,

*‘The community has the fear of assisting those in need for fear of getting infected with COVID-19.’* (SU19, Kenya)

*‘There’s a bit of mistrust in the community because everyone was afraid.’* (SU50, Benin)

Survey participants were asked a question regarding fear of creating a burden on the system, to which 24 participants (33%) responded. Several respondents were clear in their view of ‘*No because the Government should help us*’ (SU71, Vietnam), or noted that funded support care mediated the sense of burden, ‘*I felt I was a burden to my family but as [support] workers are paid to be here I don’t feel like a burden*’ (SU65, Australia). Others commented on the capacity of the system to manage any additional burden, noting ‘*The system is already poor*’ (SU04, Indonesia; SU19, Kenya).

Parents and carers of AT users were faced with difficult decisions to ensure basic needs such as food were met, for example one family who could not conduct their farming and supervise their two disabled daughters,

*We decided to move to the farms where we did all the activities there and we came back at the evening, we had to lock L and her young sister inside because we couldn’t bring them there.* (SU45, Tanzania)

Respondents commented on apparent gaps between community activities generally, and those which were inclusive of persons with disabilities with a general

*‘lack of political will to be disability inclusive in their COVID-19 program.’ *(SU52, Malawi)

*There were not much activity in the communities or in the village that included person with disability.* (SU62, Fiji)

*The community stopped including me or I feel left out when they don’t understand the impact of the pandemic towards hard of hearing person. Like the use of pen and paper. They are lazy to write and never understand that pen and paper is my way of communication.* (SU57, Philippines)

For some, disability-specific local rules and regulations cut community options off,

*Even during times when restrictions had eased for the wider community, I still felt excluded as I had to follow strict policies because of my disability and the Government rules about group living.* (SU66, Australia)

Through the lens of exclusion, COVID-19 public health measures could be seen to have both intended and unintended consequences for AT users. **Physical distancing** or social distancing means keeping a safe space between yourself and other people who are not from your household [41]. Overwhelmingly, respondents (n = 57, 78%) shared the negative impacts of social distancing on their health and wellbeing. Observing social distancing was a new experience, ‘First time in my life I have to make an effort to stay away from people’ (SU15, South Africa) and one that ‘just became a new reality of daily life’ (SU06, 64, UK). Levels of isolation increased during COVID-19,

*Due to social distancing, (he) couldn’t play outside. The situation affected (his) mental health.* (SU53, Bangladesh)

A further complication arose where social distancing cut across the strategies people used to manage daily life, that social distancing is ‘*Not possible at home and at work because I needed assistance… self-isolation is difficult because I am living with my husband and the house help in the same house*’ (SU19, Kenya) and yet seen as necessary ‘*Social distancing was mandatory to prevent the spread of COVID-19*’ (SU21, Nepal). This was particularly true for individuals with physical impairment,

*I need someone to hold me when I stand. This made me not able to be supported by someone when I stand up due to social distancing. My helper couldn’t come close to me.* (SU52, Malawi)

Participants described difficulties in managing the incompatibility of interacting with others whilst maintaining social distancing, and their efforts to self-advocate in the face of blanket policies which did not address their circumstances.

**Self-isolation** encompassed some or all of the following directives: that people must not leave their home or isolation location, except in an emergency or to get essential medical care, must not go into public places including work and shops, and must not let any other person into their home unless the person lives there and cannot live somewhere else. Forty respondents (55%) provided information about their isolation experiences. Self-isolation brought negative personal wellbeing impacts in terms of mental health,

*[Self isolation had a] clear impact on my quality of life-I had a letter from the Government to self isolate and am trying to follow this-but it is hard to be positive especially in the last 2 months! I have not been out of the house for a year now!* (SU13, UK)

Self-isolation caused lost opportunities to socialize, learn, support each other and maintain friendships for older persons,

*Sometimes, I miss my friends from another village and even from the same village. As I am mostly 90 years old, my daughter doesn’t want me to go outside during COVID-19 to protect me from the virus.* (SU42, Myanmar)

For children, loss of education and recreation opportunities significantly narrowed social networks,

*The hardest thing for E was to (not) go to class because he likes to socialize with other children, but as a measure of the family we decided not to send them to their classes anymore.* (SU46, Nicaragua)

*The game center where he met many friends was closed down, resulting in him being left alone most of the times.* (SU68, Zimbabwe)

**Lockdown**, that is, the restriction of travel, was identified by 52 respondents (71%). Lockdown severely curtailed choices, and in many instances was strongly policed,

*We have strict guidance so this framed my daily life choices.* (SU06, UK)

*In COVID time fear is in every person or in society. Lockdown imposed and police not allowed anyone to go out for anything. No one supported us because we are living in remote area in small village.* (SU14, India)

From the perspective of AT users and their families, although lockdown rules changed over the course of months and rules differed, there appeared to be no public health awareness of the needs of AT users, or sensitivity in terms of the policy response.

*…it was locked down family that lost employment to support person with disabilities-had issues in buying diapers, etc.* (SU62, Fiji)

*Q could not go to the provincial hospital or annual health check and getting new arm brace during COVID-19.* (SU72, Vietnam)

Across both low- and high-income countries, habilitation and development goals were delayed or lost,

*There were many restrictions in the area (travel, transportation, crowd gathering, etc.). This has caused the centre that the child is attending to have closed… socio-educational support is made difficult by the absence of a sign language facilitator, because the little girl does not use articulated language.* (SU36, Benin)

Table 1 summarizes a range of impacts of restrictions described by AT users and their families. Resilience and positivity in the face of lockdown, physical distancing and self-isolation restrictions were also evident. Respondents described positives among the negative impacts, for example unexpected contact and support from neighbors, or the use of virtual meeting places, ‘*improved hygiene in the home, personalized attentions, meeting new families through social networks*’ (SU37, India). A number of children reported enriched family and home environments, with everyone staying together,

*During isolation at home my father and brother spent more time with me.* (SU40, Cameroon)

*His parents stayed at home and spent more time to teach (him).* (SU71, Vietnam)

Policy mandates for **personal protective equipment** **(PPE)** raised issues of access, of compliance, of unexpected consequences, and of affordability for 57 respondents (78%). People often wished to comply as these rules were seen as sensible, ‘*Good way to prevent the spread of COVID-19*’ (SU08) and often mandatory, ‘*we were forced to buy the mask before going inside the hospital*’ (SU33). However, the cost burden of purchase of personal protective equipment was a substantial barrier,

*People with disabilities were not a high priority to get PPE, or even considered, sometimes.* (SU61, Australia)

*Yes like mask, soap and sanitizer, but we could not managed to afford because of poor economic status.* (SU45, Tanzania)

A specific group of AT users for whom these public health measures held specific negative consequences were users of AT for hearing who relied upon visual communication,

*The social distancing impacted my services negatively. When I communicated with people wearing masks, I had to lean closer to the people to hear and understand what they were saying. In a normal scenario, I would lip-read and communicate. In addition, I had to deal with background noise. However, if I stood close enough, I would be able to understand the other person, despite the mask. Considering the COVID-19 guidelines (social distancing), this is probably a Catch-22 situation.* (SU12, India)

*Masks made it difficult for me to lip read as people wore masks over their mouth, and I sometimes had to request them to remove their masks while maintaining social distancing which often wasn’t comfortable.* (SU10, India)

While a number of respondents withdrew and stayed isolated due to difficulties in adhering to PPE requirements, a range of alternatives and workarounds were actively pursued by many. Civil society and individual efforts stepped in to enable access, ‘*Access to masks was difficult at one time. … the Centre had to manufacture them in-house and distribute them to children and parents, As well as handwashing kits*’ (SU39, Tanzania).

Others, particularly users of hearing and respiratory AT recognized the importance of masks to keep each other safe, however, sought alternative solutions to standard masks to meet their need to communicate,

*Masks has been helpful. However, it causes communication barrier. If only transparent facemasks are allowed to be worn.* (SU57, Philippines)

A subtheme of exclusion was that of **creating inclusion**. Respondents were asked what qualities or attributes about themselves, or their family helped them manage the impacts of COVID-19; what organizations did that helped throughout this time; and how our health and support systems could be better prepared in the future.

All participants answered one or more of these questions, identifying personal and family resilience and endurance, followed by community and NGO support, and lastly government support, as having helped in managing the impacts of COVID-19,

*Good neighbors and family love sharing our resources together. *(SU34, Cameroon)

*Psycho-social support of volunteers.* (SU05, Ukraine)

*Family solidarity…Disabilities Organizations in Rwanda keep me inform(ed) and involve me in helping others.* (SU26, Rwanda)

*We have supported our neighbors in this COVID time because we are living in remote area in small village. One time we have received food from local government officials.* (SU24, India)

Resilience was a key theme for some, who felt living with functional impairments meant they had already developed a degree of resilience in the face of isolation, exclusion and occupational deprivation, and were able to share this lived experience with others,

*Because everyone is feeling socially isolated now (not just me), they are moving to this. So the burden of dealing with social isolation doesn’t fall just on me, as its more equal, the burden is shared, and I don’t just feel its me and my needs making this happen.* (SU61, Australia)

Some examples of inclusion emerged and, in some instances, met unmet need and promised improved community access in contrast to pre-COVID-19 opportunities,

*There are some times when the people from health… come to ask for persons with disabilities for inclusion on some COVID relief programs.* (SU68, Zimbabwe)

*He had unmet need for a hearing device pre COVID. This was provided thanks to an inclusive COVID-19 response program and will transform his difficulties hearing and communicating during his social activities. However, due to COVID-19, social activities have been abolished and Mbah W feels unsafe going outside.* (SU16, Indonesia)

Two quotes from respondents of the Western Pacific (Fiji) and African regions summarize the vision for an AT inclusive pandemic response,

*Health and support systems should have a preparedness plan that gives a special focus to poor families with a member(s) with disabilities prior to incidents such as COVID-19… When there is no such plans, in times like these when we compete for limited resources, our type of families are easily forgotten in everything.* (SU44, Tanzania)

*Consultation with disability sector planning and ways forwards so when it done it’s done for all.* (SU62, Fiji)

These reported impacts of the pandemic on communities in which AT users live, and the consequences of public health pandemic responses, evidence a need to understand and mitigate against the impact health responses may have on people who use AT, and to consult with civil society including AT users, their families and representative bodies in order to make pandemic public health responses inclusive of people who use AT.

**Communication** was a strong theme in creating inclusion. Consistent with the population at large, respondents commented that COVID-19 information was not sufficiently timely, clear, or fully disseminated. For many AT users, this was compounded by lack of accessible information formats. Communication issues fell into two parts: lack of information regarding COVID-19 (28 responses or 38%), and lack of information about changes in how assistive products and services can be accessed (38 responses or 52%). Overwhelmingly, participants felt they lacked information on accessing assistive products and services due to COVID-19 related changes, as one respondent observed, ‘*It was difficult to think through what the general restrictions meant for my particular services, and often they didn’t know either*’ (SU61, Australia). Table 2 outlines common difficulties and solutions in the words of respondents, with proposed communication principles for more inclusive public health messaging summarised in Column 3.

A final subtheme of creating inclusion concerned **enabling technologies**. For those with access to telecommunication products and infrastructures, online and virtual connection opportunities sprang up and, in part, appeared to meet community inclusion needs, ‘*We have a Whats App group now if we need it-before we didn’t know each other*’ (SU13, UK).

Communication technology became a dominant enabler of community, social and civic life, with particular benefits for AT users with mobility and energy restrictions,

*I feel included in social activities that are now available to me online and can be viewed flexibly when I’m awake, instead of at set times when I am too tired. *(SU61, Australia)

Education, leisure and work was described as taking other occupational forms, enabled by internet access and virtual connection,

*I love to play table tennis and enjoy life to the fullest (now) participation has become all online activities.* (SU23, India)

*All the services that were meaningful to me were all closed. However, my exercise physiologist and my drama group started to offer video link appointments or group activities. I did not have the technology or internet to access these, and I needed help to learn how to use this… I was still able to participate in my drama group sessions with my classmates, friends and trainers through a virtual environment. It is undoubtedly true that this iPad and internet connection, has become an important part of my daily activities since the beginning of the pandemic. I believe having a stable and economical internet plan is essential for me to fully participate in meaningful activities and essential services.* (SU66, Australia)

Some respondents described new opportunities which this created, for example learning new skills in the online environment,

*The self-isolation impacted me positively because I could explore calls via Zoom and Teams. Using Teams, Zoom, and headphones enables me to focus more on my speech, and understand people clearly. Online calls have actually proved to be an advantage because I must rely on just one sense-hearing. This has made me more sensitive and empathetic to others’ voices, honing my abilities along the way.* (SU13, UK)

One AT user described his experiences of isolation and his role in supporting others,

*This hit me hard because I’m a people person. I love people. For instance, one of the girls in the service is in a home, and I ring her once a week: because it helps her and it helps me. I feel better after talking to her on the phone.* (SU55, Ireland)

However, a digital divide was evident where AT users required specific accessibility features. Lockdown closure of specialized services providing communication partners, absence of community-based rehabilitation (CBR) workers due to social distancing, and the inaccessibility of school-based AT cut some respondents off from the ability to communicate with others,

*(She) was not able to use computer at home, (had) untrained caregivers …had a lot of communication problem because of the absence of special communication board.* (SU60, Tanzania)

For digital technologies to be an enabler, hardware must be available, infrastructure such as internet access must be reliable, and users needed some level of capability.

*Q and her family members stayed at home during the social distancing period. She studied online from home. Her school updated lessons to students via parents mobile phone.* (SU72, Vietnam)

The following example illustrates how a student workforce were able to work with an AT user to seek alternate forms of social and community interaction, however, the cost of ICT infrastructure was an issue,

*Occupational therapy student input was all online, and the students did lots of work identifying options for streaming for interests. I was also able to access a live Facebook stream of puppy raising … Technology reliant on internet connection was an issue as I am on a low income and could not afford a high data plan.* (SU65, Australia)

Some AT users had specific needs in relation to digital technologies. Respondents living with hearing loss described exclusion they experienced with a shift to an online environment,

*During COVID I reduced my participation in [classroom competitions] as it becomes a bit difficult to communicate effectively over a call as compared to communication in person.... Access to good internet service helped, also, I am a persistent person, I don’t give up easily.* (SU10, India)

The critical role of AT and related supports was recognised, for example an older woman who experiences hearing loss and who coordinates a Muslim women’s group in the UK states,

*I have tried to keep my Muslim Group going as many really wanted support. Possibly no one really thought how much we rely on AT until we have these restrictions—it certainly made me think once my hearing started to deteriorate; I just need to be patient to get the new equipment. With hearing aids and the hearing clinic I can run my home, engage in discussions and online meetings with my hearing aids-otherwise I would not be able to be active.* (SU06, UK)

The opportunity to rebuild and perhaps improve systems through co-creation between AT service users and service providers was also identified as an empowering outcome,

*Last March, I felt isolated but soon after we had the virtual service running so it wasn’t that bad, but it took a lot of figuring out, together: staff and service owners worked on this together.* (SU55, Ireland)

Taken together, these data suggest that inclusive pandemic public health responses must include accessible communication formats, and recognition of information and communication technologies as priority assistive products.

#### 3.2.2. AT Services Not Seen as Essential

The COVID-19 pandemic disrupted the interrelated systems of supports and supply networks upon which people relied, and severely limited participation opportunities. Previously unmet needs were exacerbated, and new needs arose related to public health compliance,

*(COVID-19 related restrictions have) influenced my life. First by taking the others away from me a little, then the closure of the border…destroyed my mother’s small trade that allowed her to take care of my basic needs.* (SU50, Benin)

*…we were hearing from the radio, that we were advised to wash our hands with running water and for us we couldn’t able to manage it because we don’t have a source of water and for our neighbors that had water they were not allowing us to fetch it because they were afraid of contacting the disease.* (SU45, Tanzania)

*Daily life is very stressful (she) can no longer creep on the floor for fear of touching the virus. (she) need(s) a wheelchair or [crutches] that can keep (her) from the floor. (She) can no longer play with friends at school. No more visits to the physiotherapist. We needed mask and hand sanitizers. (she) was afraid going out. We are afraid of visiting the hospital.* (SU34, Cameroon)

Respondent narratives such as these demonstrate the precarious balance of paid and family support, AT provision and maintenance, and environmental facilitators such as public transport and inclusive communities. 

Lack of assistive product service delivery to address servicing and maintenance, changing needs, or growth, was identified by many participants pre-COVID and this **unmet need** was exacerbated during COVID-19 due to service restrictions and closures. Forty respondents (55%) feared being denied access to services and 51 respondents (70%) experienced actual service closure. Many respondents had poor access to services pre-COVID, and for others, fragile infrastructures such as online access or transport logistics, limited their options when services were physically closed. It is unsurprising, therefore, that 35 (48%) feared an inability to travel to the place providing products and services; and 10% of respondents feared their products may be taken away. Where there was a possibility to access healthcare, anxiety influenced people’s choices,

*This really affected G and our life in the way that we could not receive the services that we wanted. We wanted wheelchair but up to now we have not received, we wanted repair/maintenance of CP Chair but we did not receive timely service. G needs regular medication in the hospital but we received so little attention from health staff from April-July 2020. We also restricted our movements to hospitals in fear of COVID-19.* (SU39, Tanzania)

While people expressed concern and distress at the **loss of services**, and noted the adverse outcomes of service closure, no one questioned the need for such actions, with many comments regarding the serious circumstances causing closures, ‘*The local health center in M. was closed down due to COVID reported cases among health care providers’ (SU68,* Zimbabwe). Inability to access assessment, to have appropriate AT fitted, provided and maintained, led to serious participation restrictions for many,

*My daily life is so complicated because the facilities I use are not friendly e.g., normal books with small font size, untrained personnel and un recommended baby walker for mobility purposes.* (SU60, Tanzania)

*I couldn’t able to go for hearing aid repair = I couldn’t able to hear (so) Since I couldn’t able to hear family and myself struggle in communicating day-to-day activities. I have to manage on physical prompting which takes very long time to understand.* (SU43, India)

#### 3.2.3. Underlying Systems

**Figure 6:**
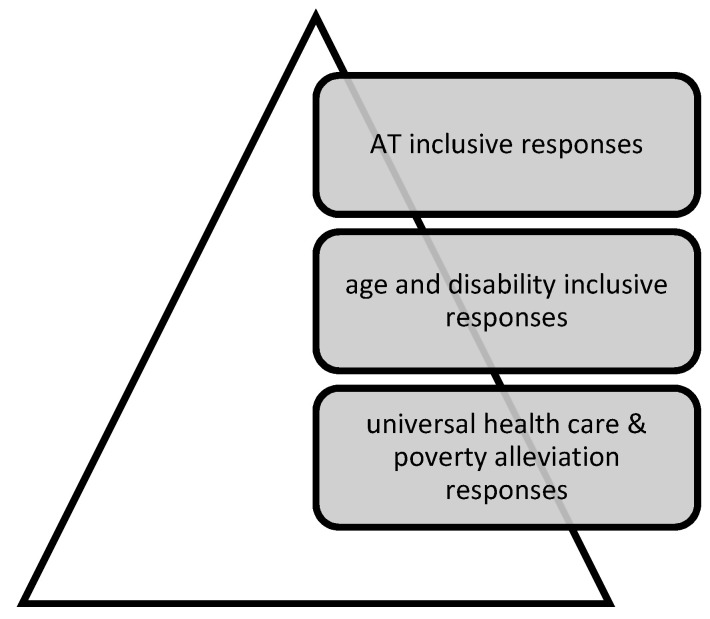
Multilevel actions required in a pandemic response.

In terms of preparing health and support systems for the future, 37 respondents (50%) offered suggestions across three levels and building from an underpinning general social safety net through to general and specific diversity considerations (Figure 6). Of these, 23 comments (62%) focused on foundation needs for universal health care and basic food and income security; 22 (59%) specified disability and ageing inclusion measures; and 10 (27%) focused on the needs of AT users.

Over half of the suggestions (20, or 54%) called for change at two or more levels of this hypothetical pyramid. Proposed solutions bundled multiple elements together,

*Income generation support to parents, repair of hearing aid. Accessibility of assistive devices, transportation, livelihood support.* (SU43, India)

*Improve accessibility and affordability of medical health and services, including the assistive products and especially for the older persons with disabilities.* (SU19, Kenya)

Taken together, proposals comprised basic food security and primary healthcare provision measures; disability inclusive strategies such as data collection on the needs of people with disability and older persons to enable priority responses; and nuanced responses at the AT systems level to meet the provision, use, repair and replacement needs of users of diverse assistive products.

Turning specifically to AT systems, Table 3 describes examples of difficulties they experienced in sustaining access to AT services and solutions. Proposed principles for sustaining services are summarized in Column 3.

The data described granular issues with AT services across many settings and countries, which, taken together, suggest that AT services need to be kept open, safe and accessible alongside other essential services; and that strategies are needed to increase the number and range of AT personnel, their access to training and PPE, and implementation of telehealth and other methods that enable services to continue during pandemic response measures such as isolation, social distancing and/or lockdowns.

## 4. Discussion

**Figure 7:**
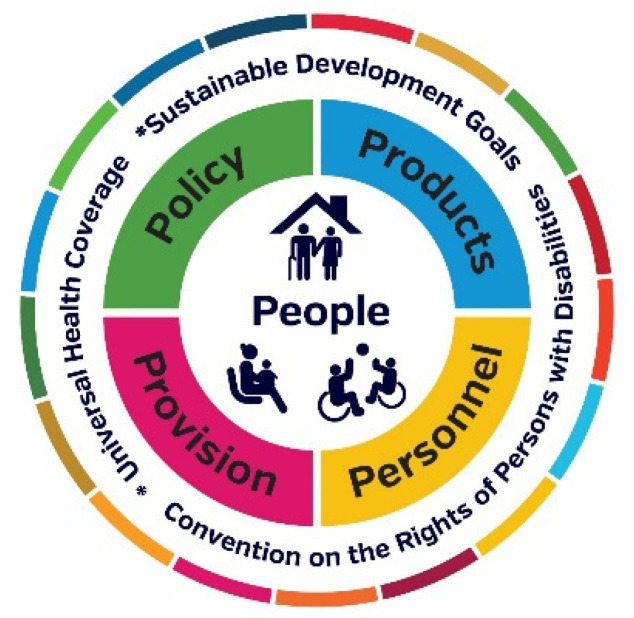
WHO GATE 5 P Framework.

Our findings illuminate the experiences and perspectives of AT users and their families from 24 countries across the six WHO Regions during the COVID-19 pandemic, focused on the period of January 2020–March 2021. Recruitment resulted in balanced gender, and approximately equal cohorts of young people and adults, with slightly fewer older persons. Efforts to balance participants across world regions were less successful, and some regions had extremely low response rates of three and less (Eastern Mediterranean and Americas) while others had higher response rates of twenty and over (South East Asia and Africa). The majority of countries were in low- and middle-income brackets, and, while participants were not asked to specify urban, regional or remote setting, narratives illuminated a range of settings. Mindful of these limitations, the experiences and perspectives captured do provide opportunities to review and strengthen AT systems across the WHO GATE 5P Framework [12] (Figure 7) for now and the future.

The 5 P framework is used to discuss study data and relevant literature and to contextualize the study recommendations, presented below.

### 4.1. People

Older people and people with disability make up the two largest groups of people who use AT. This study data and current evidence demonstrates that AT users experience additional trauma related to living through the COVID-19 pandemic, including social grief, isolation and intersectional stressors [42], and fear of economic insecurity and healthcare rationing [11,26,43,44]. In their review of COVID-19 studies across low- and middle-income countries and inclusive of older persons living with disability, Banks et al identify that ‘People with disabilities will be disproportionately affected by the economic implications of the ongoing COVID-19 pandemic unless responses are disability-inclusive’ [45], and our data illustrates this clearly. The key theme emerging from the data to address and alleviate this vulnerability concerns comprehensive, affordable and inclusive two-way communication pathways, presented in Table 2. The literature echoed the importance of digital capability and related AT for communication and accessible health messaging [28], for provision of AT services, and for social connection while physically distanced [23,46,47].

### 4.2. Policy

Research on pandemic policy finds that governments issue inconsistent policy narratives [48] based on ambiguous priorities [11]. Every jurisdiction we sampled had implemented some form of public health policy measure, often reinforced by legislation and subject to policing. Without exception, AT policy principles across the globe were subsumed by the rapid, changeable and often untested deployment of public health policy designed to address COVID-19. Our evidence suggests that pandemic-management policy has been designed around normative assumptions of a majority population, not addressing the diverse realities of AT users, including older people, people with disabilities, and those living with chronic illnesses. A range of literature calls for individual and systemic action to address ‘systemic and institutional ableism’ [42] evident through the medical rationing of resources such as ventilators and supplies [49] and similar breaches of disability justice [11]. AT users and their families call for age and disability inclusive COVID-19 responses to meet the needs of AT users.

### 4.3. Products

Focusing on AT use during COVID-19, a series of recent studies found AT policies and systems are inadequate to prevent service disruption, a lack of prioritization of AT, unaffordability, failure to meet complex needs, problems with infection control, insufficient emergency preparedness, and lack of AT or provider availability [46,47]. The data presented by AT users and families in Table 3 speak to many of these concerns, and envisions workarounds and innovations to address these shortfalls.

### 4.4. Personnel

Critical discussions are occurring in the literature regarding how health services might be transformed given pandemic restrictions [50,51], and how health professions may respond [50,51,52]. Data from this study provided some perspectives from AT users and families as to the capability and responsiveness of AT personnel (see other studies in this series) as well as the impacts of the loss of other personnel (community-based rehabilitation workers, volunteers, school staff, community members) upon their health, development, and wellbeing.

### 4.5. Provision

The literature illustrates that COVID-19 has impacted the foundations of health and security for people living with disabilities and the impacts of ageing in specific ways, and these findings resonate with our data. Measures to contain the spread of COVID-19, as well as increased pressure on health systems, have resulted in significant disruptions for AT users, with a key contributing factor being the distance to centralized services. AT provision can be supported by family efforts, through virtual social networks, and by replacing face to face activities with tele-enabled activities [30,53]. However, there are risks for vulnerable populations such as AT users of accentuated health disparities as clinic-based services switch to telemedicine [10], an issue of concern to some of our respondents without reliable or affordable connectivity.

The following recommendations are made based on this discussion (Table 4):

In summary, the imperative for an AT-inclusive pandemic response is demonstrated throughout our data and the resulting recommendations represent a key addition to the evidence base. AT-inclusive pandemic preparedness and response goes beyond current calls for disability inclusive pandemic preparedness and response. Current disability inclusion evidence clearly identifies the disproportionate risk of increased morbidity and mortality [23], the need for enhanced disability justice in health care settings, and the need for policy transparency and accountability in rationing approaches [11]. Our study illuminates additional dimensions of experience which have, to date, not been made explicit in disability inclusion literature, related to life stage. We refer to the developmental, educational and growth-related imperatives for young and very young AT users [24]. We also refer to the specific needs and vulnerabilities of older people [54].

## 5. Conclusions

Understanding the lived experience of AT use and service delivery before and during the COVID-19 pandemic informs a critical reflection on the effectiveness of government strategies and of civil society responses. Pre-pandemic, AT service access was difficult to access for many, and AT user narratives identify constraint-induced service provision innovations which can address disruption due to this and future emergency situations. These emerging alternate models of AT service provision are less reliant upon tertiary level, centralized services; and rather, capitalize on existing health system infrastructure, technology advancement and task sharing to bring AT services closer to people’s homes.

Recommendations for AT systems strengthening are as follows. Pandemic responses must be AT-user inclusive, and that the developing concept of disability-inclusive policy must encompass this additional dimension within its scope. AT must be recognized as essential health products and services, and AT service delivery strengthened to ensure it reaches those who need it. Consultation with civil society including AT users and their families, providers, and representative organizations is essential. Our evidence demonstrates the importance of the WHO GATE programmatic response, and points to a set of recommendations to protect and uphold the rights of AT users in pandemic and disaster preparedness and response.

## Figures and Tables

**Table 1:** Impacts of restrictions.

Negative Impacts	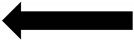	Aspect of Life during COVID-19	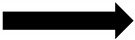	Positive Impacts
Felt unsafe going outside Worried people around me may contract COVID-19 At risk of contracting COVID-19 Fear of becoming a burden on the system Fear my products might be taken away for use by others		**Life During a Pandemic**	Previously unmet needs addressed in response ‘People were kinder’ People living with impairments prepositioned to be resilient when faced with constraints	
Services closed Denied access to AT services No information about how to recieve AT help Could not travel to places providing AT services		**Lockdown**	Creative service delivery Skilling of AT users via online modes Co-creation of workable online services Physical access barriers to community/cultural activities reduced as these events became virtual	
Less human connection, loneliness Loss of face-to-face services and personal supports led to feeling isolated, losing abilities, and development delays in children		**Physical Distancing**	Opportunity to reskill, e.g., deaf persons in online environments Emergence/strengthening of online communities Emergence/strengthening of different service delivery models including online services	
A need for new equipment to be safe, like masks My community stopped including me Mask-wearing prevented communication Hygiene products too expensive or not available		**Personal Protective Equipment**	Creative solutions to mask wearing Sense of self-efficacy in disease prevention	

**Table 2:** Communication difficulties, solutions, and lessons.

AT Users’ Perspectives on Public Health Messaging		Communication Principles for More Inclusive Public Health Messaging
Difficulties	Solutions	
**Timing and complexity:** ‘*There is a lot but it is changing quickly, so feel a bit confused at times*’ (SU13, UK) *I see the communication is so muddled and confusing-so needs to be clearer guidance. See the messaging needs to be clearer for us all and also to be able to share our concerns and give suggestions*. (SU13, UK) **Accessibility:** *(accessible (braille) materials are not available for reading on COVID)* (SU54, India) *…not in a format that can be used by all disabilities* (SU52, Malawi) *It wasn’t translated in my local language* (SU70, Zimbabwe) **AT service specific information:** *Lack of information about changes in how assistive products and services can be accessed* (SU16, Indonesia) *The support systems should inform us to stock up on hearing aid batteries, and in case of a faulty hearing aid, the support system should provide us with a contact number so that the hearing aid can be repaired or replaced* (SU12, India)	**Regular, prompt and local:** *Information received through daily media briefs by the Ministry of Health* (SU19, Kenya) *The village administrator explained deeply about COVID-19 news and preventive measures … daily by using loudspeaker* (SU42, Myanmar) *In our living area, local authorities promptly update information about the COVID pandemic, which helps us to keep aware of protecting our family* (SU72, Vietnam) **Access and accessibility:** *I have a television set and a radio: therefore, getting information especially through radio in vernacular language *(SU03, Kenya) *(need to) improve the use of visual audio media and social networks* (SU27, Nicaragua) **Accuracy:** *Give to the people the truth about COVID-19 and how people can immunize themselves. Give to people accurate information and give them basic knowledge in health issues especially persons with disabilities* (SU26, Rwanda)	Make use of multiple and diverse opportunities, platforms and networks to disseminate regular, reliable and transparent public health messages across local areas and languages. Public health messages should be accessible for all including people with vision, hearing, cognition or communication access needs. Provide specific messaging for people who use AT regarding how changes will impact them and actions they can take.

**Table 3:** AT service access difficulties, and solutions.

Perspectives of People Who Use AT on Service Access		Principles for Sustaining AT Services
Examples of Difficulties	Examples of Solutions	
**Access to services and service personnel:** *Assessment (was) remote, then access to therapies with extreme protective and health measures…but the remote follow-ups were inoperative* (SU37, Nicaragua) *Many businesses were closed and it was difficult to get an appointment* *and if you did-do you really want to go?* (SU18, Kenya) *There was restriction in accessing service centres and I had also fear of getting infected* (SU53, Bangladesh) *My orthotics need to be renewed because I have grew up a little bit* (SU50, Benin) *Rehabilitation services … were stopped hence no services related to assistive devices and services were rendered* (SU45, Tanzania) *I was not allowed to go to some services, and many couldn’t come to me. Physio, OT students and OT, and masseuse weren’t allowed to visit* (SU65, Australia) **Issues of supply and repair:** *I can’t control on my urine and stool, so I have to use urine bag every time but in COVID time we were face many problem in getting it* (SU27, India) *The lockdown caused hearing aid centers to close and thus, we can’t avail the services and maintenance for our hearing aids. We can’t even buy batteries* (SU57, Philippines) *N’s hearing aids couldn’t be repaired so she refused to wear it sometimes as the family couldn’t adjust in a right way* (SU67, Vietnam) *I used to go center for repair and maintenance and sometimes as follow up once or twice in a month… Due to locked down it was closed for certain times until the service provider got safety measures* (SU21, Nepal)	**Telehealth and regular contact:** *... so I used zoom where possible but it was not always suitable* (SU65, Australia) *I was in touch with service provider through telecommunication so when the situation was under control I got access to service center* (SU21, Nepal) *The telehealth policy was only for rural people but has been expanded to everybody and this has helped a lot. Its worrying that this might go away afterwards* (SU61, Australia) *I used e-appointments and video-calling for minor prosthetic issues* (SU23, India) *I can access online and if anything needs to be sent to me, {Provider] organize for it to be delivered. If I just need advice, AT are on the end of the phone* (SU55, Ireland) *I was in touch with service provider through telecommunication so when the situation was under control I got access to service center *(SU21, Nepal) **Stocking up:** *During COVID-19, I ensured that I stocked up on hearing aid batteries* (SU12, India) **AT Services opened with protection measures:** *Services are open but ‘must follow health safety protocol*’ (SU72, Vietnam)	Use telehealth including zoom consultations and telephone contact to bridge service gaps, support regular contact between AT users and service providers and reduce access barriers. Where internet and delivery services operate well, online ordering systems can support access to consumables and spare parts. AT services require access to training for personnel and equipment to ensure adequate infection control measures, reducing the risk of face-to-face contact for all involved.

**Table 4:** Recommendations.

Recommendations
**Make pandemic public health responses inclusive of people who use AT:** Consult with civil society including AT users, their families and representative bodies Understand and mitigate against the impact health responses may have on people who use AT Use communication formats that ensure public health messages are accessible to all, including people with hearing or vision loss, or disabilities that impact cognition and/or communication Recognize information and communication technologies including smart phones, as priority assistive products. **Recognize AT as essential health products and services and during a pandemic or health emergency:** Keep AT services open, safe and accessible alongside other essential services Consult with and include AT personnel in health sector wide responses Provide AT personnel with infection control training and personal protective equipment Implement telehealth and other methods that enable services to continue during pandemic response measures such as isolation, social distancing and/or lockdown Prioritise continued procurement and supply of quality-assured assistive products **Strengthen AT services to improve preparedness for future pandemic responses:** Integrate AT services into health care systems and in particular community/primary health care Address access barriers and increase coverage through outreach visits, telehealth and other strategies Train and equip a broader range of health personnel able to provide and/or support AT use

## Data Availability

All relevant data has been supplied in the article. Individual responses received will behave been stored securely as per ethical approval.

## Appendix A. Rapid Evidence Review

A rapid evidence review sourced relevant peer-reviewed literature. Two authors ((NL; IC) trialled several iterations of keyword searches (assistive technology; assistive products; COVID-19; older people/person; disability; ageing; survey) with the final search string [assistive technology and COVID-19] run in PubMed and Google Scholar.

Given the rapid emergence of COVID-19 relevant literature, searches were run at two time points within the research project (October 2020; March 2021) The yield from the initial search informed development of the survey tool. A small number of additional records from the second search.

In total, n-662 de-duplicated titles and abstracts were screened, with n-52 full text records reviewed. A final yield of n-40 studies provided a rapid review of current literature, which was used to develop the survey question set as well as informing the discussion and recommendations.

## Appendix B

**Table A1:** Survey Form for AT User/Families.

About Me	
My name and the place I live:	Free text responses
If you choose, tell us about you and your life	
**Life before COVID-19**	
**List the assistive products and services that you used before COVID-19**	
Before COVID-19, were there other supports that you needed that you didn’t have?	
Before COVID-19, did you have any difficulty accessing assistive products and services?	
**The next questions are about your participation before COVID-19** **Before COVID-19, did you:**	
Take care of yourself?	
Do tasks at home, for yourself and others?	
Participate in other activities *(Examples: work, study, fun, community)*	
Access assistive technology services *(Examples: Assessment, maintenance, repairs, training)*	
**Life now, during COVID-19**	
**What months did COVID-19 impact your access to services? Tick all that apply**	
**2020** January; February; March; April; May; June; July; August; September; October; November; December **2021** January; February; March	
**List the assistive products and services that you used now, during COVID-19** *Examples: Individual supports (e.g., assistive products or home adaptations): Support people (e.g., caregivers, family, paid helpers): Societal supports (e.g., accessible transport)*	
**What is your daily life like with these supports?**	
**Do you need any other supports to live your daily life**	
**Are there any barriers to accessing assistive products and services?**	
**During COVID-19, what is different about how you:**	
Take care of yourself	
Do tasks at home, for yourself and others?	
Participate in other activities * (Examples: work, study, fun, community)*	
Access assistive technology services * (Examples: Assessment, maintenance, repairs, training)*	
**What do you think most impacted your services? *(either in a good or bad way)***	
Social distancing	
Self-isolation	
The region where I live was locked down	
The services I use were closed	
I was at risk of contracting COVID-19	
Important people around me were contracting COVID-19	
There was a need for new equipment to be safe, like masks	
I didn’t feel safe going outside	
My community stopped including me	
Fear of being denied access to services	
Fear my products might be taken away for use by others (for example, a ventilator or other respiratory device)	
Lack of information about COVID	
Lack of information about changes in how assistive products and services can be accessed	
Fear of creating a burden on the system	
Inability to travel to the place providing products and services	
Other	
**Addressing the challenges**	
**What qualities/attributes about you and your family, have helped you manage the impacts of COVID-19?**	
**What have other organisations done that has helped you throughout this time?** *Example: civil society organisations, government?*	
**What do you think could have been helpful, but was not done?**	
**How could our health and support systems be better prepared in the future?**	
